# Endoscopic Sphincterotomy Prior to Biliary Stenting for Malignant Biliary Obstruction: A Systematic Review and Meta‐Analysis of Randomized Controlled Trials

**DOI:** 10.1111/jgh.70714

**Published:** 2026-09-04

**Authors:** Sofia Coelho Meine, Gabriel de Oliveira Amaral, Mateus Rech Tedesco, João Pedro Schmitt, Gabrielle Knackfuss D'Aiuto, Gilmara Coelho Meine

**Affiliations:** ^1^ Feevale University Novo Hamburgo Rio Grande do Sul Brazil

**Keywords:** biliary stenting, endoscopic sphincterotomy, malignant biliary obstruction, pancreatitis

## Abstract

**Background and Aims:**

ERCP‐guided biliary stenting is the usual palliative treatment for patients with unresectable malignant biliary obstruction (MBO). Previous studies suggested that ES prior to biliary stenting may not reduce the post‐ERCP pancreatitis (PEP) risk and may increase bleeding risk. This systematic review and meta‐analysis aimed to evaluate the effectiveness and safety of ES prior to biliary stenting in patients with MBO, with a particular focus on the impact of MBO etiology and stent type.

**Methods:**

We systematically searched for randomized controlled trials (RCTs) comparing ERCP‐guided biliary stenting with versus without ES in patients with MBO. We estimated the pooled risk ratios (RRs) with respective 95% confidence intervals (CIs) using a random‐effects model.

**Results:**

Six RCTs (1215 patients) were included. Technical success and the risk of PEP, clinically significant bleeding, perforation, cholangitis, cholecystitis, and stent malfunction were similar between the groups. Subgroup analyses by MBO etiology and stent type were consistent with overall analysis, except for PEP by MBO etiology. ES reduced PEP risk in studies with a low prevalence of pancreatic cancer (RR 0.23; 95% CI 0.12–0.44), but no significant difference was observed in studies with a high prevalence of pancreatic cancer (RR 0.85; 95% CI 0.57–1.28).

**Conclusion:**

The risk of clinically significant bleeding was similar with or without ES prior to biliary stenting for MBO. ES was associated with a reduced PEP risk in studies with a low prevalence of pancreatic cancer, whereas no benefit was observed in studies with a high prevalence of pancreatic cancer.

## Graphical abstract

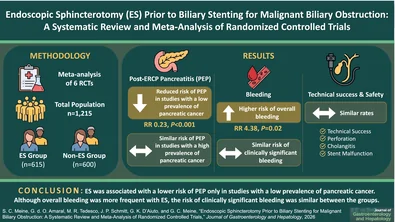

**Abbreviations**
CI: confidence interval
ERCP: endoscopic retrograde cholangiopancreatography
ES: endoscopic sphincterotomy
GRADE: Grading of Recommendations, Assessment, Development, and Evaluation
MBO: malignant biliary obstruction
NSAID: nonsteroidal anti‐inflammatory drug
PEP: post‐ERCP pancreatitis
PRISMA: Preferred Reporting Items for Systematic Reviews and Meta‐Analyses
PROSPERO: International Prospective Register of Systematic Reviews
RCTs: randomized controlled trials
RR: risk ratios
SEMS: self‐expandable metal stents

## 1 Introduction

Endoscopic retrograde cholangiopancreatography (ERCP)‐guided biliary stenting is the usual palliative treatment for patients with unresectable malignant biliary obstruction (MBO), relieving symptoms and improving survival [1]. Despite its frequent use and high technical success rates, ERCP‐guided biliary stenting is associated with clinically relevant adverse events, particularly post‐ERCP pancreatitis (PEP), bleeding, perforation, cholangitis, and cholecystitis [2].

Although endoscopic sphincterotomy (ES) is often performed to facilitate stent placement, a prior systematic review suggested that ES does not reduce PEP risk and may increase bleeding risk [3]. However, the clinical impact of ES may vary according to the etiology of MBO and the type of stent used [4, 5]. Therefore, further investigation is warranted to better define the role of ES across different clinical scenarios.

This systematic review and meta‐analysis aimed to evaluate the effectiveness and safety of ES prior to biliary stenting in patients with MBO, with a focus on the impact of MBO etiology and stent type on outcomes.

## 2 Methods

### 2.1 Protocol and Registration

We conducted and reported this systematic review and meta‐analysis in accordance with the Cochrane Handbook for Systematic Reviews of Interventions and the Preferred Reporting Items for Systematic Reviews and Meta‐Analyses (PRISMA) statement [6, 7]. We prospectively registered the study protocol in the International Prospective Register of Systematic Reviews (PROSPERO; CRD42026136).

### 2.2 Search Procedure and Eligibility Criteria

We searched MEDLINE, Embase, and Cochrane Library up to April 2026. Table S1 describes the search strategy. The inclusion criteria were: (1) full‐text, peer‐reviewed RCTs; (2) adult patients with MBO; (3) comparison of ERCP‐guided biliary stenting with versus without ES; and (4) availability of data for at least one prespecified outcome. No language restrictions were applied. We excluded studies in patients with benign biliary obstruction or non‐ERCP‐guided biliary stenting, observational studies, non‐randomized trials, editorials, literature reviews, letters to the editor, or conference abstracts.

### 2.3 Outcomes and Subgroup Analyses

The primary outcome was PEP. The secondary outcomes included successful stent insertion, overall bleeding, clinically significant bleeding, perforation, cholangitis, cholecystitis, and stent malfunction (stent migration or stent occlusion). Overall bleeding included all bleeding events reported in the original studies. Clinically significant bleeding was predefined as unplanned hospital admission or prolongation of hospitalization, need for blood transfusion, or need for repeat therapeutic endoscopy or radiologic intervention. Bleeding classified as moderate or severe by Cotton criteria [8] or the American Society for Gastrointestinal Endoscopy Lexicon [9] was also considered clinically significant because these severity categories are consistent with our predefined definition. Stent malfunction included stent migration or occlusion.

We were uncertain whether the etiology of MBO and the type of biliary stent could influence the relative treatment effects. Therefore, we conducted subgroup analyses according to MBO etiology (studies with < 60% vs. ≥ 60% of patients with pancreatic cancer) and biliary stent type (self‐expandable metal stents [SEMS] vs. plastic stents).

### 2.4 Study Selection and Data Extraction

Two reviewers (G.O.A. and M.R.T.) independently conducted the search using Zotero software (Version 6.0.26, Corporation for Digital Scholarship, Vienna, VA, USA). After excluding duplicates, they screened the titles and abstracts. Subsequently, the eligibility of each article was assessed through a full‐text review. Backward snowballing was performed using the reference lists of included RCTs and previous systematic reviews and meta‐analyses. A third author (G.C.M.) resolved disagreements.

For each included trial, we collected information on study characteristics (title, authors, year of publication, and country), study design, sample size, follow‐up duration, use of nonsteroidal anti‐inflammatory drug (NSAID), participant characteristics (age, sex, and MBO etiology), ERCP‐guided biliary stenting strategy (with or without ES, and the type of stent used [SEMS or plastic stent]), and outcomes of interest. Means and standard deviations were estimated from medians and ranges, according to Wan et al. [10] and Luo et al. [11].

### 2.5 Quality Assessment

Two independent reviewers (G.K.D. and J.P.S.) assessed the risk of bias using the Cochrane Risk of Bias 2 tool [12]. We derived the overall bias risk assessment for each trial from individual domain judgments. We could not perform a publication bias assessment through funnel plots because fewer than 10 studies were available for inclusion in the meta‐analysis [13]. For each outcome, we evaluated the certainty of the evidence using the Grading of Recommendations, Assessment, Development, and Evaluation (GRADE) tool [14], classifying it as high, moderate, low, or very low certainty.

### 2.6 Data Analyses

We used a random‐effects model to estimate pooled risk ratios (RRs) and corresponding 95% confidence intervals (CIs) for dichotomous outcomes. A *p*‐value < 0.05 was defined as statistically significant. Between‐study heterogeneity was assessed using Cochran's Q test and quantified with the *I* ^2^ statistic; *p*‐values < 0.10 for Cochran's Q test and *I* ^2^ ≥ 25% were considered significant for between‐study heterogeneity. If significant heterogeneity was detected, we conducted leave‐one‐out sensitivity analysis. We performed the prespecified subgroup analysis if ≥ 2 studies contributed data to each subgroup. A *p*‐value *<* 0.10 was considered statistically significant for subgroup interaction. We conducted the statistical analyses using Review Manager 5 (Version 5.4, The Cochrane Collaboration, Copenhagen, Denmark).

## 3 Results

The initial search identified 345 records. After removing duplicates and screening titles and abstracts, we reviewed the full‐text of eight studies and included six RCTs [15, 16, 17, 18, 19, 20] (Figure 1). The included studies encompassed 1215 patients, of whom 615 (50.62%) were randomized to the ES group. Pancreatic cancer accounted for more than 65% of MBO cases in four studies [15, 16, 18, 20], whereas in the remaining two studies [17, 19], pancreatic cancer accounted for less than 45% of cases. Four studies [16, 17, 18, 20] used SEMS, and the remaining two studies [15, 19] used plastic stents. Baseline characteristics of the included RCTs are described in Table 1. The studies excluded during full‐text screening and the corresponding reasons for exclusion are listed in Table S2.

**FIGURE 1:**
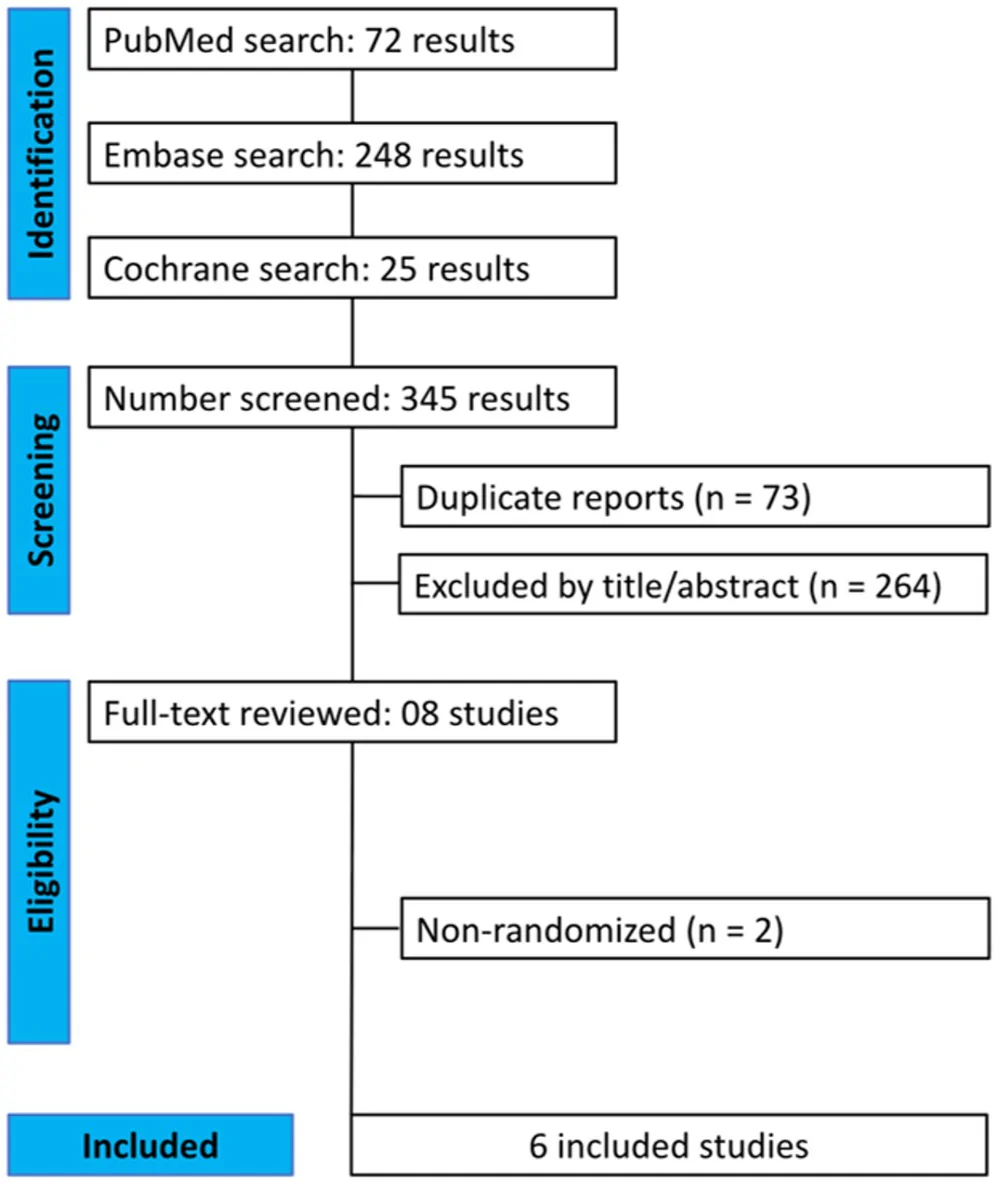
PRISMA flow diagram of study screening selection.

**TABLE 1:** Baseline characteristics of included studies.

First author, year	Country	Design	Stent type	Follow‐up, days		Sample size		Male, *n* (%)		Mean age, years (SD)		NSAID use, *n* (%)		Pancreatic cancer, *n* (%)	
				ES	Non‐ES	ES	Non‐ES	ES	Non‐ES	ES	Non‐ES	ES	Non‐ES	ES	Non‐ES
Giorgio, 2004 [13]	Italy	RCT single‐center	Plastic	115^a^		96	96	51 (53.1)	47 (48.9)	72^b^	75^b^	NA	NA	64 (66.6)	67 (69.8)
Artifon, 2008 [14]	Brazil	RCT multicentric	PCSEMS	132^a^	159^a^	37	37	18 (48.7)	12 (32.4)	72.1	65.4	NA	NA	30 (81.1)	30 (81.1)
Zhou, 2012 [15]	China	RCT single‐center	USEMS	180	180	41	41	24 (58.5)	23 (56.1)	65.1 (9.5)	64.0 (7.5)	NA	NA	11 (26.8)	10 (24.4)
Hayashi, 2015 [16]	Japan	RCT multicentric	PCSEMS	180	180	100	100	53 (53.0)	49 (49.0)	72.2 (10.6)	70.5 (10.4)	NA	NA	100 (100)	100 (100)
Kato, 2022 [17]	Japan	RCT multicentric	Plastic	30	30	185	185	112 (60.5)	112 (60.5)	70.8 (10.5)	70.9 (9.6)	51 (27.6)	54 (29.2)	83 (44.9)	81 (43.8)
Onnekink, 2025 [18]	Netherlands and Spain	RCT multicentric	FCSEMS	30	30	156	141	86 (55.1)	74 (52.5)	70^b^	69^b^	151 (96.8)	133 (94.3)	111 (71.2)	108 (76.6)

Abbreviations: ES, endoscopic sphincterotomy; FCSEMS, fully‐covered self‐expandable metal stent; NA, not available; NSAID, nonsteroidal anti‐inflammatory drug; PCSEMS, partially‐covered self‐expandable metal stent; RCT, randomized controlled trial; SD, standard deviation; USEMS, uncovered self‐expandable metal stent.

^a^ Mean.

^b^ Median.

### 3.1 Primary Outcome

All included studies [15, 16, 17, 18, 19, 20] assessed PEP risk in patients with MBO, and no statistically significant difference was identified between the groups undergoing ERCP‐guided biliary stenting with versus without ES (RR 0.53; 95% CI 0.26–1.08; *p* = 0.08; *I* ^2^ = 69.6%; Figure 2; Table 2). In the leave‐one‐out sensitivity analyses, excluding Kato et al. [19] reduced heterogeneity and did not significantly impact the effect estimate (RR 0.73; 95% CI 0.45–1.18; *I* ^2^ = 19.7%; Table S3).

**FIGURE 2:**
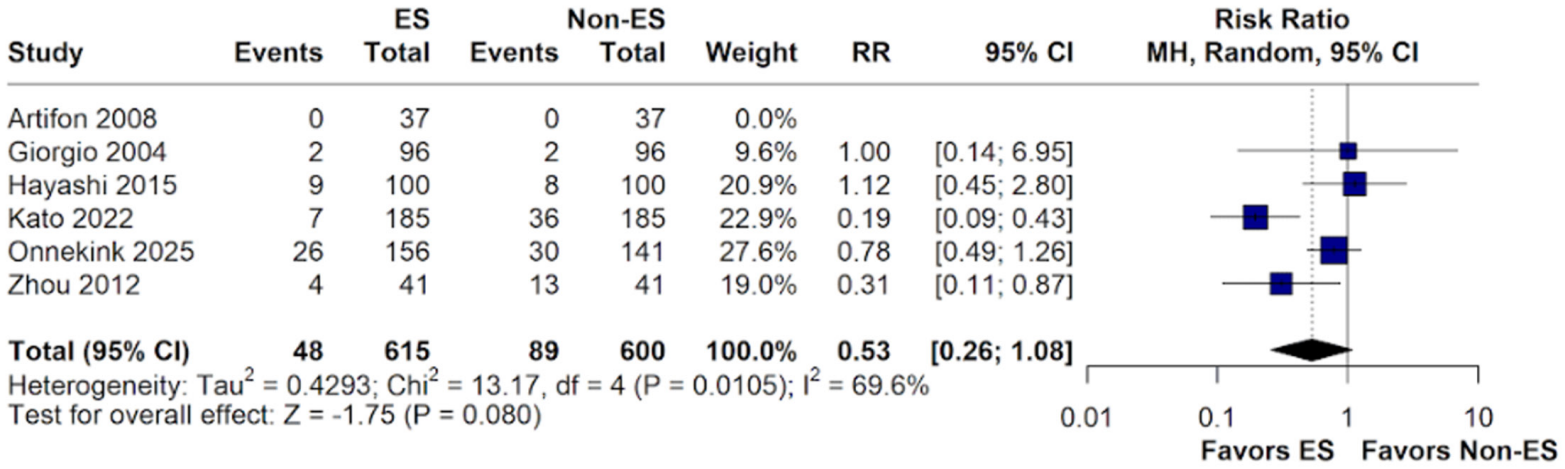
Forest plot of post‐ERCP pancreatitis. CI, confidence interval; ES, endoscopic sphincterotomy; MH, Mantel–Haenszel.

**TABLE 2:** Summary of findings and certainty of evidence.

Outcome	Studies (patients)	Risk ratio (95% confidence interval)	*p*	*I* ^2^	Certainty of evidence
Post‐ERCP pancreatitis	6 RCTs (1215)	0.53 (0.26–1.08)	0.08	69.6%	⨁⨁⨁◯ Moderate^a^
Overall bleeding	5 RCTs (1119)	4.38 (1.29–14.89)	0.02	0%	⨁⨁⨁◯ Moderate^b^
Clinically significant bleeding	5 RCTs (1119)	2.47 (0.42–14.70)	0.32	22%	⨁⨁⨁◯ Moderate^b^
Perforation	4 RCTs (927)	1.06 (0.11–10.50)	0.96	39.8%	⨁⨁◯◯ Low^a^, ^b^
Stent malfunction	6 RCTs (1215)	0.97 (0.69–1.36)	0.85	0%	⨁⨁⨁⨁ High
Cholangitis	5 RCTs (1127)	1.14 (0.82–1.59)	0.42	23.6%	⨁⨁⨁⨁ High
Cholecystitis	3 RCTs (853)	0.70 (0.24–2.10)	0.53	40.7%	⨁⨁◯◯ Low^a^, ^b^
Technical success	6 RCTs (1215)	1.00 (0.99–1.01)	0.92	0%	⨁⨁⨁⨁ High

Abbreviations: ERCP, endoscopic retrograde cholangiopancreatography; RCTs, randomized controlled trials.

^a^ Inconsistency (significant heterogeneity).

^b^ Imprecision (small sample size or wide confidence interval).

### 3.2 Secondary Outcomes

In patients with MBO, ERCP‐guided biliary stenting with ES was associated with a higher overall bleeding risk (RR 4.38; 95% CI 1.29–14.89; *p* = 0.02; *I* ^2^ = 0%; Figure 3A; Table 2) than biliary stenting without ES. However, the clinically significant bleeding risk was similar between the groups (RR 2.47; 95% CI 0.42–14.7; *p* = 0.32; *I* ^2^ = 22%; Figure 3B; Table 2).

**FIGURE 3:**
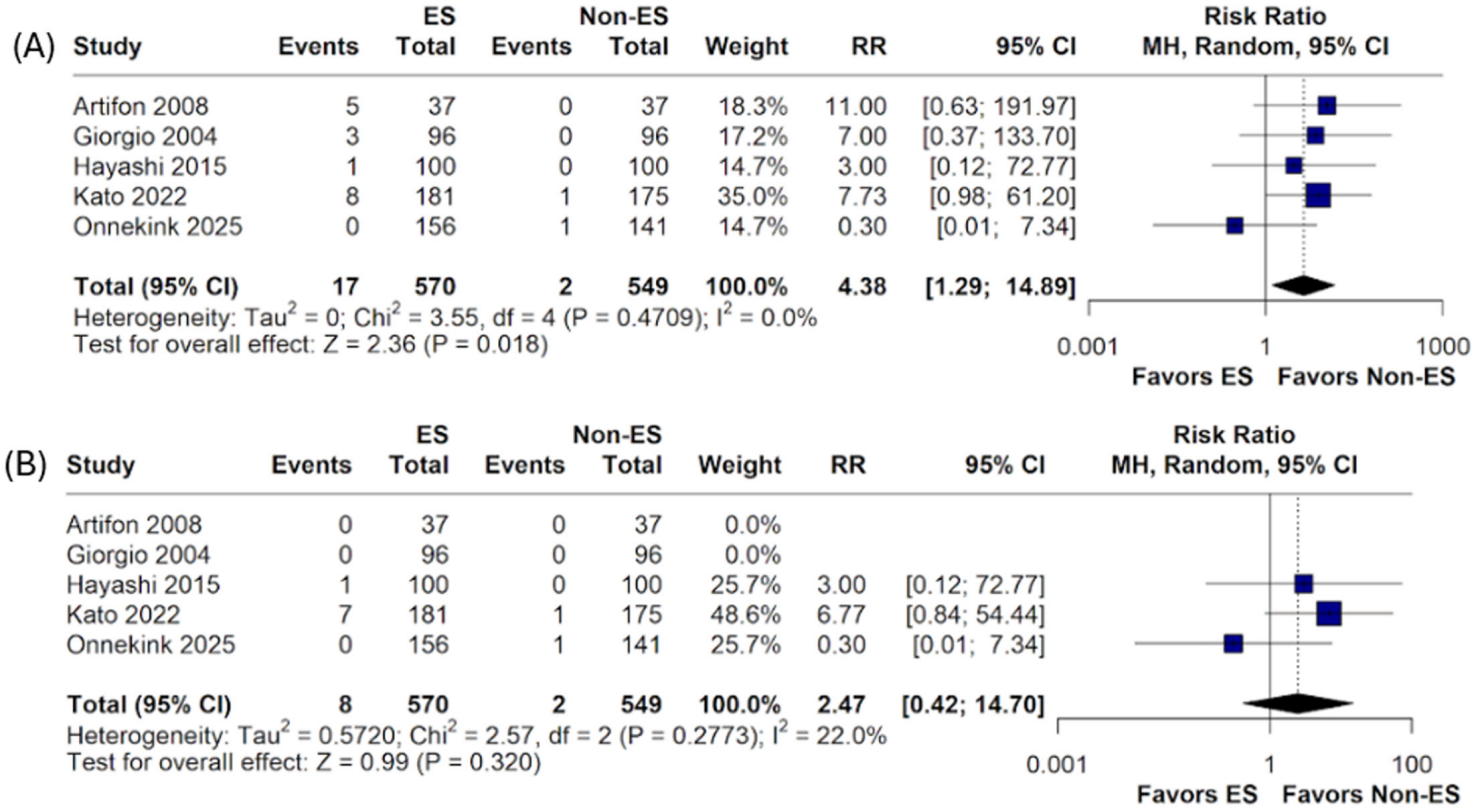
Forest plot of (A) overall bleeding and (B) clinically significant bleeding. CI, confidence interval; ES, endoscopic sphincterotomy; MH, Mantel–Haenszel.

Both groups showed a comparable probability of technical success (RR 1.00; 95% CI 0.99–1.01; *p* = 0.92; *I* ^2^ = 0%; Figure S1; Table 2). Similarly, there was no significant difference in the risk of perforation (RR 1.06; 95% CI 0.11–10.50; *p* = 0.96; *I* ^2^ = 39.8%; Figure S2; Table 2), stent malfunction (RR 0.97; 95% CI 0.69–1.36; *p* = 0.85; *I* ^2^ = 0%; Figure S3; Table 2), cholangitis (RR 1.14; 95% CI 0.82–1.59; *p* = 0.42; *I* ^2^ = 23.6%; Figure S4 Table 2), and cholecystitis (RR 0.70; 95% CI 0.24–2.10; *p* = 0.53; *I* ^2^ = 40.7%; Figure S5; Table 2).

In the leave‐one‐out sensitivity analysis, the exclusion of Artifon et al. [16] eliminated the heterogeneity in the outcome of perforation (RR 0.32; 95% CI 0.03–3.03; *I* ^2^ = 0%; Table S3), and the exclusion of Hayashi et al. [18] reduced the heterogeneity in cholecystitis (RR 1.00; 95% CI 0.39–2.57; *I* ^2^ = 7%; Table S3), without significantly impacting the effect estimate.

### 3.3 Additional Analyses

There were no significant subgroup differences based on the etiology of MBO (studies with < 60% vs. ≥ 60% of patients with pancreatic cancer) or the type of biliary stent (SEMS vs. plastic stents) (Figures S6–S13), except for PEP according to MBO etiology, which showed significant treatment interaction (test for subgroup differences: *p* = 0.0006; Figure 4). In studies in which pancreatic cancer accounted for < 60% of cases, ERCP‐guided biliary stenting with ES was associated with a reduced PEP risk (RR 0.23; 95% CI 0.12–0.43; *p* < 0.001; *I* ^2^ = 0%). In contrast, among those studies with ≥ 60% pancreatic cancer, the PEP risk was similar between the groups (RR 0.85; 95% CI 0.57–1.28; *p* = 0.45; *I* ^2^ = 0%). Importantly, this subgroup analysis eliminated heterogeneity within both subgroups.

**FIGURE 4:**
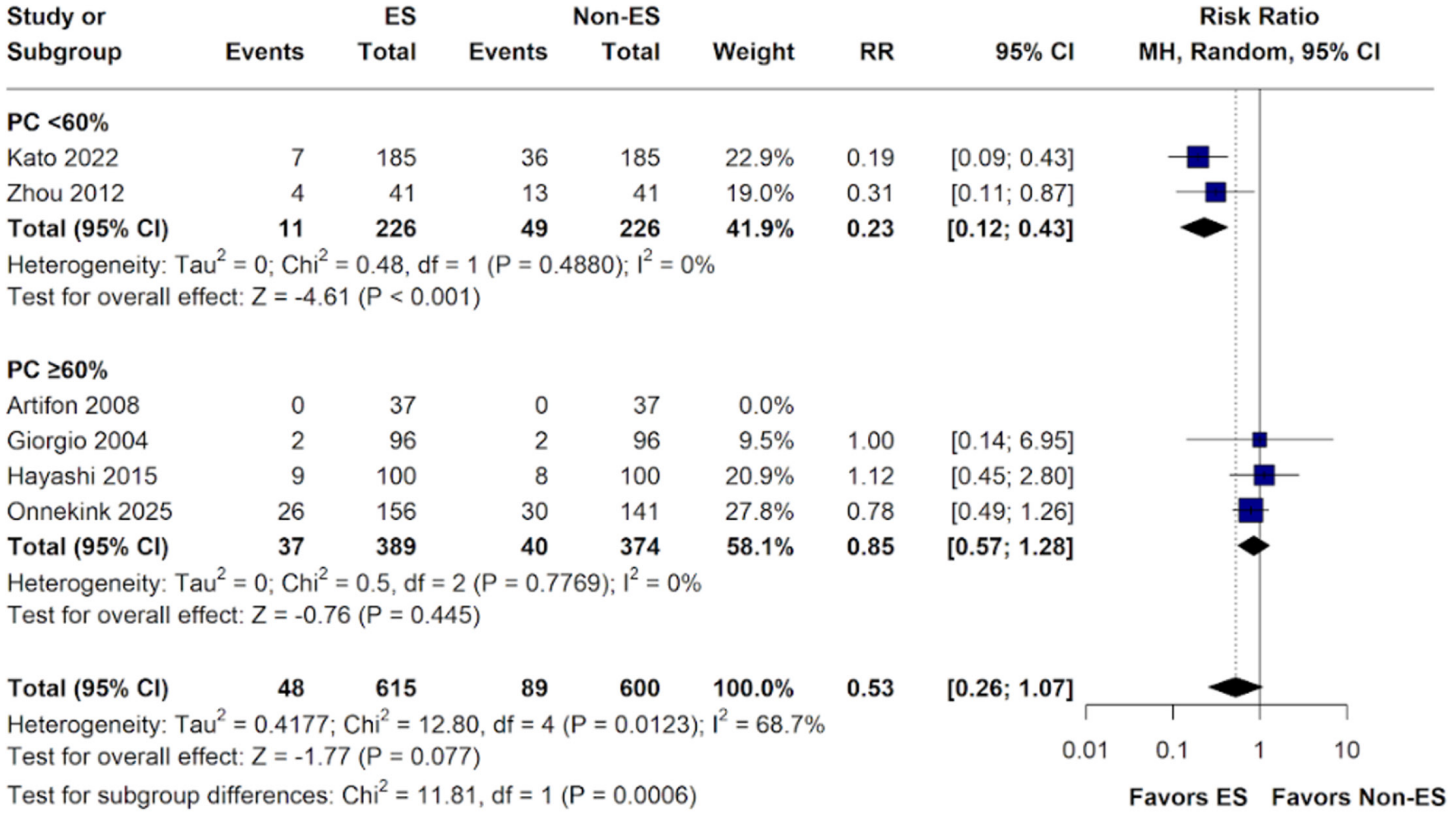
Subgroup analysis of post‐ERCP pancreatitis according to the etiology of malignant biliary obstruction. CI, confidence interval; ES, endoscopic sphincterotomy; MH, Mantel–Haenszel; PC, pancreatic cancer.

### 3.4 Quality Assessment

The results of the risk of bias assessment according to the Cochrane Risk of Bias 2 tool are described in Figure S14. Three trials [15, 16, 17] raised concerns, mainly due to the risk of bias in the selection of reported results. All other RCTs showed a low risk of bias [18, 19, 20]. The summary of our findings and the overall certainty of the evidence according to the GRADE tool are presented in Table 2.

## 4 Discussion

This systematic review and meta‐analysis comprised over 1200 patients with MBO and showed a similar risk of PEP between groups. However, subgroup analysis by MBO etiology indicated that ES was associated with a reduced PEP risk in studies with lower pancreatic cancer prevalence, whereas no benefit was observed in studies with high pancreatic cancer prevalence. ERCP‐guided biliary stenting with ES was associated with a higher overall bleeding risk than biliary stenting without ES, but the risk of clinically significant bleeding was similar between the groups. Technical success and the risk of perforation, cholangitis, cholecystitis, and stent malfunction were also similar between groups.

Although the overall analysis showed similar PEP risk between groups, the subgroup analysis by MBO etiology indicated that ES was associated with reduced PEP risk in studies with lower pancreatic cancer prevalence, while no significant difference was observed in studies with high pancreatic cancer prevalence. These findings are supported by Tarnasky et al. [4], who demonstrated that patients with proximal biliary strictures had a significantly higher risk of pancreatitis after biliary stenting, whereas no cases of pancreatitis were observed among patients with distal strictures. The authors proposed that proximal strictures may create a “fulcrum effect,” leading the stent to deflect medially and compress the pancreatic orifice, increasing the risk of pancreatic duct obstruction and pancreatitis. In contrast, distal MBO, which is commonly caused by pancreatic cancer, may be associated with a lower baseline risk of PEP because these patients frequently have chronic pancreatic duct obstruction and pancreatic atrophy.

A previous meta‐analysis evaluating biliary stenting for MBO with versus without ES found that ES was associated with a higher overall bleeding risk [3], consistent with our findings. However, we observed that only 10 of the 19 bleeding events reported in the included studies (52.6%) met criteria for clinically significant bleeding, and the risk of clinically significant bleeding did not differ between groups. This distinction is clinically important because minor bleeding events are often self‐limited and rarely require intervention. Additionally, in patients undergoing SEMS placement for MBO, fully covered self‐expandable metal stents (FCSEMSs) can be used as an effective rescue therapy for severe post‐sphincterotomy bleeding, with hemostatic success rates exceeding 90% [21].

Our findings should be interpreted considering the temporal differences across studies over more than 20 years, during which ERCP techniques evolved, rectal NSAIDs became standard prophylaxis for PEP, SEMS technology improved, and the definition and reporting of adverse events became increasingly standardized. For instance, several earlier studies did not report the use of prophylactic NSAIDs for PEP prevention, whereas Kato et al. [19] reported NSAID use in approximately 28% of patients and Onnekink et al. [20] in nearly all patients (> 95%). Importantly, among studies reporting use of NSAIDs, prophylactic strategies were balanced between treatment groups, which may have minimized the risk of confounding related to PEP prevention practices.

The World Endoscopy Organization guideline on ERCP biliary cannulation and sphincterotomy techniques recommends that routine ES should not be performed before biliary stent placement for MBO but highlights the need for further investigation in the specific setting of transpapillary FCSEMS placement in patients without chronic pancreatic duct obstruction [22]. Our subgroup analysis provides evidence relevant to this uncertainty, showing ES was associated with a reduced PEP risk in studies with a lower prevalence of pancreatic cancer, whereas no benefit was observed in studies predominantly including patients with pancreatic cancer, possibly reflecting differences in the location of the obstruction and the presence of chronic pancreatic duct obstruction or pancreatic atrophy. However, because pancreatic cancer prevalence was evaluated at the study level and may correlate with other clinical characteristics, these findings should be considered hypothesis‐generating. They support patient‐level trials stratified by the etiology of MBO and incorporating potential effect modifiers such as stricture location, pancreatic duct obstruction, and pancreatic atrophy.

This study has some strengths. This is the largest meta‐analysis of RCTs evaluating ES prior to biliary stenting for MBO. Furthermore, the included trials were conducted across several geographic regions, improving the generalizability of the findings to diverse clinical settings.

Our meta‐analysis also has several limitations. First, the number of included trials was relatively small. Second, between‐study heterogeneity was observed for some outcomes. However, leave‐one‐out sensitivity analyses identified the main sources of heterogeneity, and exclusion of these studies did not significantly impact the effect estimates. Additionally, heterogeneity was reduced or eliminated in subgroup analyses stratified by MBO etiology and stent type. Third, subgroup analyses were based on study‐level rather than patient‐level data and should be interpreted cautiously. Finally, the absence of patient‐level data and relevant procedural variables, such as use of rectal NSAID prophylaxis, difficult cannulation, pancreatic duct manipulation, and operator experience, prevented a more definitive assessment of the patient and procedural characteristics that may modify the relative efficacy and safety of ES in this setting.

In conclusion, ES prior to biliary stenting was consistently associated with an increased overall bleeding risk but had no significant impact on the risk of clinically significant bleeding. The etiology of MBO appears to modify the risk of PEP because ES was associated with a reduced PEP risk in studies with low pancreatic cancer prevalence, whereas no benefit was observed in studies with high pancreatic cancer prevalence. Further patient‐level studies that stratify outcomes according to the underlying etiology of MBO and incorporate potential effect modifiers are warranted to confirm these findings.

## Funding

The Article Processing Charge for the publication of this research was funded by the Coordenação de Aperfeiçoamento de Pessoal de Nível Superior ‐ Brasil (CAPES).

## Disclosure

All authors take responsibility for the reliability and freedom from bias of the data presented and their interpretation.

## Conflicts of Interest

The authors declare no conflicts of interest.

## Supporting information


**Table S1:** Search strategy.
**Table S2:** List of excluded studies at the full‐text screening stage and the reasons for exclusion.
**Table S3:** Leave‐one‐out sensitivity analysis.
**Figure S1:** Forest plot of technical success. CI, confidence interval; ES, endoscopic sphincterotomy; MH, Mantel–Haenszel.
**Figure S2:** Forest plot of perforation. CI, confidence interval; ES, endoscopic sphincterotomy; MH, Mantel–Haenszel.
**Figure S3:** Forest plot of stent malfunction. CI, confidence interval; ES, endoscopic sphincterotomy; MH, Mantel–Haenszel.
**Figure S4:** Forest plot of cholangitis. CI, confidence interval; ES, endoscopic sphincterotomy; MH, Mantel–Haenszel.
**Figure S5:** Forest plot of cholecystitis. CI, confidence interval; ES, endoscopic sphincterotomy; MH, Mantel–Haenszel.
**Figure S6:** Subgroup analysis of stent malfunction according to the etiology of malignant biliary obstruction. CI, confidence interval; ES, endoscopic sphincterotomy; MH, Mantel–Haenszel; PC, pancreatic cancer.
**Figure S7:** Subgroup analysis of cholangitis according to the etiology of malignant biliary obstruction. CI, confidence interval; ES, endoscopic sphincterotomy; MH, Mantel–Haenszel; PC, pancreatic cancer.
**Figure S8:** Subgroup analysis of technical success according to the etiology of malignant biliary obstruction. CI, confidence interval; ES, endoscopic sphincterotomy; MH, Mantel–Haenszel; PC, pancreatic cancer.
**Figure S9:** Subgroup analysis of post‐ERCP pancreatitis according to the type of biliary stent. CI, confidence interval; ES, endoscopic sphincterotomy; MH, Mantel–Haenszel; SEMS, self‐expandable metal stent.
**Figure S10:** Subgroup analysis of bleeding according to the type of biliary stent. CI, confidence interval; ES, endoscopic sphincterotomy; MH, Mantel–Haenszel; SEMS, self‐expandable metal stent.
**Figure S11:** Subgroup analysis of stent malfunction according to the type of biliary stent. CI, confidence interval; ES, endoscopic sphincterotomy; MH, Mantel–Haenszel; SEMS, self‐expandable metal stent.
**Figure S12:** Subgroup analysis of cholangitis according to the type of biliary stent. CI, confidence interval; ES, endoscopic sphincterotomy; MH, Mantel–Haenszel; SEMS, self‐expandable metal stent.
**Figure S13:** Subgroup analysis of technical success according to the type of biliary stent. CI, confidence interval; ES, endoscopic sphincterotomy; MH, Mantel–Haenszel; SEMS, self‐expandable metal stent.
**Figure S14:** Risk of bias assessment. D1, randomization process; D2, deviations from intended interventions; D3, missing outcome data; D4, measurement of the outcome; D5, selection of the reported results.

## Data Availability

This systematic review and meta‐analysis used publicly available data from the researched databases. No new datasets were generated or analyzed during the current study.
