# Supplementary material for: Endoscopic Sphincterotomy Prior to Biliary Stenting for Malignant Biliary Obstruction: A Systematic Review and Meta‐Analysis of Randomized Controlled Trials
Source: J Gastroenterol Hepatol. 2026 Sep 4;41(11):3348–55. doi: 10.1111/jgh.70714 (PMC13731063; doi:10.1111/jgh.70714)
Supplement: Supplementary file 1 [file JGH-41-3348-s001.docx]

**SUPPLEMENTARY MATERIAL**

**Endoscopic Sphincterotomy Prior to Biliary Stenting for Malignant Biliary Obstruction: A Systematic Review and Meta-Analysis of Randomized Controlled Trials**

**Authors:** Sofia Coelho Meine; Gabriel de Oliveira Amaral; Mateus Rech Tedesco; João Pedro Schmitt; Gabrielle Knackfuss D’Aiuto; Gilmara Coelho Meine, MD, PhD.

**Institution:** Feevale University, Novo Hamburgo, RS, Brazil.

**Supplementary Table S1.** Search strategy

(“malignant biliary obstruction” OR “malignant common bile duct obstruction” OR “bile duct neoplasms” OR "biliary tract neoplasms” OR “pancreatic cancer” OR “biliary stricture” OR “biliary strictures”)

AND

(“endoscopic sphincterotomy” OR “biliary sphincterotomy” OR “endoscopic biliary sphincterotomy” OR “endoscopic papillotomy”)

AND

(“self-expandable metal stent” OR “self-expandable metal stents” OR SEMS OR “plastic stent” OR “plastic stents” OR “biliary stenting”).

**Supplementary Table S2.** List of excluded studies at the full-text screening stage and the reasons for exclusion.

| **Excluded study** | **Reason for exclusion** |
| --- | --- |
| Kim HG, Han J, Kim HJ, et al. Minor endoscopic sphincterotomy to prevent post-ERCP pancreatitis before SEMS insertion for malignant biliary obstruction. Pancreas. 2014;43(8):1236–1240. | Non-randomized study |
| Nam HS, Kang DH, Kim HW, et al. Efficacy and safety of limited endoscopic sphincterotomy before self-expandable metal stent insertion for malignant biliary obstruction. World J Gastroenterol. 2017;23(9):1627-1636. | Non-randomized study |

**Supplementary Table S3.** Leave-one-out sensitivity analysis.

| **Study removed** | **Risk ratio**  **[95% confidence interval]** | **I²** |
| --- | --- | --- |
| **Post-ERCP pancreatitis** | | |
| None | 0.53 [0.26, 1.08] | 69.6% |
| Giorgio et al., 2004 | 0.49 [0.22, 1.09] | 76.7% |
| Artifon et al., 2008 | 0.53 [0.26, 0.08] | 69.6% |
| Zhou et al., 2012 | 0.60 [0.26, 1.41] | 74.2% |
| Hayashi et al., 2015 | 0.43 [0.18, 1.01] | 72.1% |
| Kato et al., 2022 | 0.73 [0.45, 1.18] | 19.7% |
| Onnekink et al., 2025 | 0.46 [0.18, 1.18] | 68.2% |
| **Perforation** | | |
| None | 1.06 [0.11, 10.50] | 39.8% |
| Artifon et al., 2008 | 0.32 [0.03, 3.03] | 0% |
| Hayashi et al., 2015 | 1.77 [0.06, 50.36] | 58.8% |
| Kato et al., 2022 | 1.06 [0.11, 10.50] | 39.8% |
| Onnekink et al., 2025 | 1.86 [0.07, 48.05] | 56.3% |
| **Cholecystitis** | | |
| None | 0.70 [0.24,2.10 | 40.7% |
| Hayashi et al., 2015 | 1.00 [0.39, 2.57] | 7.0% |
| Kato et al., 2022 | 0.53 [0.17, 1.60] | 40.2% |
| Onnekink et al., 2025 | 0.74 [0.07, 7.99] | 67.9% |

**Supplementary Figure S1.** Forest plot of technical success. CI, confidence interval; ES, endoscopic sphincterotomy; MH, Mantel–Haenszel.

**Supplementary Figure S2.** Forest plot of perforation. CI, confidence interval; ES, endoscopic sphincterotomy; MH, Mantel–Haenszel.

**Supplementary Figure S3.** Forest plot of stent malfunction. CI, confidence interval; ES, endoscopic sphincterotomy; MH, Mantel–Haenszel.

**Supplementary Figure S4.** Forest plot of cholangitis. CI, confidence interval; ES, endoscopic sphincterotomy; MH, Mantel–Haenszel.

**Supplementary Figure S5.** Forest plot of cholecystitis. CI, confidence interval; ES, endoscopic sphincterotomy; MH, Mantel–Haenszel.

**Supplementary Figure S6.** Subgroup analysis of stent malfunction according to the etiology of malignant biliary obstruction. CI, confidence interval; ES, endoscopic sphincterotomy; MH, Mantel–Haenszel; PC, pancreatic cancer.

**Supplementary Figure S7.** Subgroup analysis of cholangitis according to the etiology of malignant biliary obstruction. CI, confidence interval; ES, endoscopic sphincterotomy; MH, Mantel–Haenszel; PC, pancreatic cancer.

**Supplementary Figure S8.** Subgroup analysis of technical success according to the etiology of malignant biliary obstruction. CI, confidence interval; ES, endoscopic sphincterotomy; MH, Mantel–Haenszel; PC, pancreatic cancer.

**Supplementary Figure S9.** Subgroup analysis of post-ERCP pancreatitis according to the type of biliary stent. CI, confidence interval; ES, endoscopic sphincterotomy; MH, Mantel–Haenszel; SEMS, self-expandable metal stent.

**Supplementary Figure S10.** Subgroup analysis of bleeding according to the type of biliary stent. CI, confidence interval; ES, endoscopic sphincterotomy; MH, Mantel-Haenszel; SEMS, self-expandable metal stent.

**Supplementary Figure S11.** Subgroup analysis of stent malfunction according to the type of biliary stent. CI, confidence interval; ES, endoscopic sphincterotomy; MH, Mantel–Haenszel; SEMS, self-expandable metal stent.

**Supplementary Figure S12.** Subgroup analysis of cholangitis according to the type of biliary stent. CI, confidence interval; ES, endoscopic sphincterotomy; MH, Mantel–Haenszel; SEMS, self-expandable metal stent.

**Supplementary Figure S13.** Subgroup analysis of technical success according to the type of biliary stent. CI, confidence interval; ES, endoscopic sphincterotomy; MH, Mantel–Haenszel; SEMS, self-expandable metal stent.

**Supplementary Figure S14.** Risk of bias assessment. D1, randomization process; D2, deviations from intended interventions; D3, missing outcome data; D4, measurement of the outcome; D5, selection of the reported results.
